# Association between frequency of mass media exposure and maternal health care service utilization among women in sub-Saharan Africa: Implications for tailored health communication and education

**DOI:** 10.1371/journal.pone.0275202

**Published:** 2022-09-29

**Authors:** Richard Gyan Aboagye, Abdul-Aziz Seidu, Bright Opoku Ahinkorah, Abdul Cadri, James Boadu Frimpong, John Elvis Hagan, Nigussie Assefa Kassaw, Sanni Yaya

**Affiliations:** 1 Department of Family and Community Health, School of Public Health, University of Health and Allied Sciences, Hohoe, Ghana; 2 Department of Estate Management, Takoradi Technical University, Takoradi, Ghana; 3 Centre for Gender and Advocacy, Takoradi Technical University, Takoradi, Ghana; 4 College of Public Health, Medical and Veterinary Sciences, James Cook University, Townsville, Australia; 5 School of Public Health, Faculty of Health, University of Technology Sydney, Sydney, Australia; 6 Department of Family Medicine, Faculty of Medicine, McGill University, Montreal, Quebec, Canada; 7 Department of Social and Behavioural Science, School of Public Health, University of Ghana, Legon, Accra, Ghana; 8 Department of Health, Physical Education, and Recreation, University of Cape Coast, Cape Coast, Ghana; 9 Faculty of Psychology and Sport Sciences, Neurocognition and Action-Biomechanics-Research Group, Bielefeld University, Bielefeld, Germany; 10 School of Public Health, Addis Ababa University, Addis Ababa, Ethiopia; 11 School of International Development and Global Studies, University of Ottawa, Ottawa, Canada; 12 The George Institute for Global Health, Imperial College London, London, United Kingdom; FHI360, UNITED STATES

## Abstract

**Introduction:**

Awareness creation through mass media has the potential to promoted positive behaviors and discourage negative health-related behaviors through direct and indirect pathways. In this study, we examined the association between exposure to mass media and maternal health care services utilization among women in sub-Saharan Africa.

**Methods:**

We used data from the recent Demographic and Health Surveys (DHS) conducted between 2010 and 2020. A total of 28 countries with a survey dataset within 2010–2020 were included in our study. We included 199,146 women who had ever had a pregnancy in the last five years preceding the survey. Weighting was applied. Multilevel mixed-effect models were considered to account for cluster-level variations and correct inferences. Fixed and random effects estimates were reported. Adjusted odds ratio (aOR) with their 95% confidence intervals (CIs) were used to present the results. Also, we presented the random intercept variations, intraclass correlation coefficient, and model fitness.

**Results:**

Women who listened to radio at least once every week (aOR = 1.11, 95% CI = 1.07,1.15) were more likely to attend ANC as against those who did not listen to radio at all. Also, women who watched television at least once a week (aOR = 1.39, 95% CI = 1.33,1.46) were more likely to attend ANC compared to those who did not watch television at all. Women who read newspaper/magazine at least once a week (aOR = 1.27, 95% CI = 1.14,1.41); listened to radio at least once a week (aOR = 1.12, 95% CI = 1.07,1.17); and watched television at least once a week (aOR = 1.32, 95% CI = 1.24,1.40), were more likely to utilize SBA than those who did not read newspaper/magazine; listen to radio; and watch television at all. Women who read newspaper/magazine at least once a week (aOR = 1.35, 95% CI = 1.27,1.45); listened to radio at least once a week (aOR = 1.37, 95% CI = 1.32,1.42); and watched television at least once a week (aOR = 1.39, 95% CI = 1.32,1.47) were more likely to utilize PNC compared to those who did not.

**Conclusions:**

The study identified a strong positive relationship between mass media exposure and maternal health care services utilization. Specifically, exposure to radio and television were positively associated with ANC visitations. Moreover, exposure to mass media (newspaper/magazine, radio and television) were positively associated with SBA and PNC utilization. Policymakers and other non-governmental organizations should continuously invest resources in the design and implementation of maternal health service utilization educational programs through all the mass media channels to scale up women’s maternal health service services utilization uptake in sub-Saharan Africa.

## Introduction

There has been substantial improvement in the reduction of maternal mortality rates globally; however, sub-Saharan Africa (SSA) continues to possess a high rate of maternal mortality relative to the global front [1,2]. The World Health Organization (WHO) reported that an estimated 810 pregnant women died daily in 2017, and 94% of all maternal deaths occur in developing countries [3]. It has been widely reported that maternal health service is an important approach towards avoiding pregnancy-related complications and reducing maternal mortality in SSA [4].

Maternal healthcare is the overall wellbeing of a woman from the time of pregnancy to after birth. Maternal healthcare utilization during the three critical stages (antenatal, birth, and postnatal) is very important, as it contributes largely to reducing maternal and infant mortality and morbidity [4–6]. Antenatal care (ANC) encompasses all the routine care provided to pregnant women from conception to the onset of labor, and it helps to provide care for the prevention and management of existing and potential causes of maternal mortality and morbidity [7]. The new WHO antenatal care model recommends that the first antenatal care visits take place during the first trimester (that is below 12 weeks of pregnancy), with additional 7 visits recommended [8]. Antenatal care utilization has been reported to be key in ensuring an optimal health outcome for women and babies [9]. Skilled birth attendance (SBA) refers to pregnant women seeking care from trained health professionals to provide healthcare to mothers and newborn babies before and during delivery to manage normal deliveries and, diagnose, manage, or refer obstetric complications [10]. The use of traditional birth attendance (TBA) is predominant in most countries in SSA [11]. However, TBA is not ideal as it leads to several complications, therefore recommending SBA which reduces birth complications and maternal mortality is in the right path [12]. Postnatal care (PNC) is the care given to a mother and the newborn baby, immediately after the birth of the placenta and for the first 42 days of life [13]. A larger proportion of maternal and neonatal mortality has been reported to occur during childbirth and the postnatal period, making it a critical period for the needed health care to be available and accessed [14]. Care given at the PNC period helps health workers determine any post-delivery problems quickly and attend to them on time to prevent ill health, disability or death [15].

Given that maternal health services are important in reducing maternal mortality and morbidity, it is important that these services are utilized at each of the critical stages. To utilize these services, awareness needs to be raised on their availability and effectiveness, and mass media can be a medium for such awareness and education on maternal health services availability, importance, and effectiveness [16,17]. Mass media includes written broadcast, or spoken communication that reaches the public audience and serves as an important mechanism for societal integration [18]. It is used to disseminate information to a large audience at a relatively faster rate and at a cheaper cost [16].

Mass media promotes health through two key strategies. These strategies are by: (1) reaching a wide audience across different boundaries at the same time, and (2) exposing the public to specific messages that influence public belief, attitude, and behavior [18]. Awareness creation through mass media has the potential to encourage positive behaviors and discourage negative health-related behaviors through direct and indirect pathways [19]. Television and radio are the widely used media for creating awareness among a larger audience in SSA; nevertheless, print media such as magazine and newspaper, and outdoor media such as billboards and posters have also proven to be effective [20]. Mass media is shown to be an effective medium of reaching mothers at a large scale to enhance their utilization of maternal health services, especially in developing countries [4,19]. For example, women who read newspapers or reported watching television in Bangladesh were almost three times more likely to utilize a maternal health service [6,21]. Another study from Uganda reported a positive impact of mass media on maternal health service utilization [22].

The Sustainable Development Goals (SDG) 3.1 and 3.2 seek to reduce the global maternal mortality and end preventable deaths of newborn and under five children by 2030, respectively, with all countries targeting to reduce neonatal and under five mortality [23]. These aims are supported by other global interventions such as the strategies towards ending preventable maternal mortality [24], and the Global strategy for women’s, children’s and adolescents’ health 2016–2030 [25]. An important pillar for achieving these goals in SSA is the utilization of maternal health services. Women’s exposure to mass media (e.g., watching TV, reading a newspaper, listening to the radio, among others) can promote their utilization of maternal health services [6]. Different types of mass media may have different associations with maternal health services utilization [19]. Even though there have been some studies in SSA on the association between mass media and maternal health services utilization [26–28], there is limited literature on the association between the different types of mass media and maternal health services utilization at the SSA regional level. This study, therefore, aimed at assessing the association between the different types of mass media and maternal health services utilization among in SSA. Findings from this study could help fill an important gap in the literature on maternal health services utilization in SSA. Findings could also help in understanding the different types of mass media that can contribute to enhancing maternal health services utilization in SSA, which in turn will contribute to the reduction of maternal mortality rates in SSA and the achievement of SDG 3.1 and 3.2.

## Materials and methods

### Data source and study design

Data from the recent Demographic and Health Surveys (DHS) conducted between 2010 and 2020 were used in this study. A total of 28 countries with a survey dataset within 2010–2020 were included in our study (Table 1). The data was extracted from the women’s files of the 28 countries. DHS is a comparable nationally representative survey conducted in over 90 low-and-middle-income countries worldwide since its inception in 1984 [29]. The survey adopted a cross-sectional design to collect data from the respondents. The respondents were sampled using a two-stage sampling technique with the detailed sampling methodology highlighted in the literature [30]. The level one was women who had a pregnancy in the last five years preceding the survey and level two referred to the enumeration area or the cluster. DHS employed a structured questionnaire to collect the data on health and social indicators such as maternal health service utilization and exposure to mass media [29]. In the present study, we included 199,146 women in level one and 1611 clusters in level two. The dataset used in the study can be freely accessed at https://dhsprogram.com/data/available-datasets.cfm. We used the Strengthening the Reporting of Observational Studies in Epidemiology (STROBE) statement guidelines [31] to frame this study.

**Table 1:** Description of the sample.

Countries	Year of survey	Weighted N	Weighted %
1. Angola	2015–16	8522	4.28
2. Burkina Faso	2010	10108	5.08
3. Benin	2017–18	9101	4.57
4. Burundi	2016–17	8998	4.52
5. DR Congo	2013–14	11017	5.53
6. Congo	2011–12	5890	2.96
7. Cote d’Ivoire	2011–12	5211	2.62
8. Cameroon	2018	6666	3.35
9. Ethiopia	2016	7678	3.86
10. Gabon	2012	3658	1.84
11. Ghana	2014	4179	2.10
12. Gambia	2019–20	5415	2.72
13. Guinea	2018	5496	2.76
14. Kenya	2014	6921	3.48
15. Comoros	2012	1994	1.00
16. Liberia	2019–20	4069	2.04
17. Lesotho	2014	2597	1.30
18. Mali	2018	6671	3.35
19. Malawi	2015–16	13571	6.81
20. Nigeria	2018	22056	11.08
21. Namibia	2013	3799	1.91
22. Sierra Leone	2019	7389	3.71
23. Senegal	2010–11	6920	3.48
24. Chad	2014–15	3690	1.85
25. Togo	2013–14	4846	2.43
26. Uganda	2014–15	10231	5.14
27. Zambia	2018	7412	3.72
28. Zimbabwe	2015	5041	2.53
**All countries (SSA)**	**2010–2020**	**199,146**	**100.00**

### Variables

#### Outcome variables

Three maternal health care service utilization variables (ANC, SBA, and PNC) were the outcome variables in this study. With ANC, the women were asked the number of antenatal visits they had during the recent pregnancy. The response was continuous and was recoded into ‘No (0–3 = 0)’ and ‘Yes (4 or more = 1)’. For SBA, the women who had assistance during delivery from qualified categories of health professionals were coded as having ‘assisted delivery = 1’ whilst the remaining women were grouped as ‘not having assisted delivery = 0’. Regarding PNC, the women were asked whether they had a baby postnatal check within 2 months after delivery. The response categories were ‘No’, ‘Yes’, and ‘Don’t know’. Those who responded ‘don’t know’ were dropped. We utilized the remaining responses ‘No = 0’ and ‘Yes = 1’ in the analysis. The response coding in this study was informed by previous studies [27,28,32–34].

#### Exposure variables

Frequency of listening to radio, frequency of watching television, and frequency of reading newspapers or magazines were the key explanatory variables. All three variables had the same response options. The options were ‘not at all’, ‘less than once a week’, ‘at least once a week’, and ‘almost every day’. For this study’s purpose, those that responded, ‘at least once a week’ and ‘almost every day’ were merged and recoded as “at least once a week” and used in the study. The final response categories used in each of the three exposure variables after the recoding were “0 = not at all; 1 = less than once a week; and 2 = at least once a week”. We based on literature to code and categorize the explanatory variables [19].

#### Covariates

The covariates included in this study were selected based on their significant association with the outcome variables as well as their availability in the DHS dataset [6,19,27,28,32,33,35,36]. The variables were sectioned into individual-level factors (maternal age, educational level, religion, current working status, parity, health insurance coverage, marital status, getting medical help for self: Permission to go, getting medical help for self: distance to health facility, and getting medical help for self: getting money for treatment) and contextual factors (wealth index, place of residence, and geographical subregions). We maintained the coding for maternal age, educational level, current working status, health insurance coverage, getting medical help for self: Permission to go, getting medical help for self: distance to health facility, and getting money for treatment, wealth index, and place of residence as found in the DHS dataset. Marital status was recoded into 0 = never married; 1 = married; 2 = cohabiting; 3 = widowed; 4 = divorced; and 5 = separated. Religion was coded as 0 = Christianity; 1 = Islamic; 2 = African Traditional; 3 = No religion; and 4 = others. Parity was recoded into 0 = one birth; 1 = two births; 2 = three births; and 3 = four or more births. The 28 countries used in this study were grouped into their geographical subregions and were coded as 0 = Southern Africa; 1 = Central Africa; 2 = Eastern Africa; and 3 = Western Africa.

### Statistical analyses

**Fig 1:**
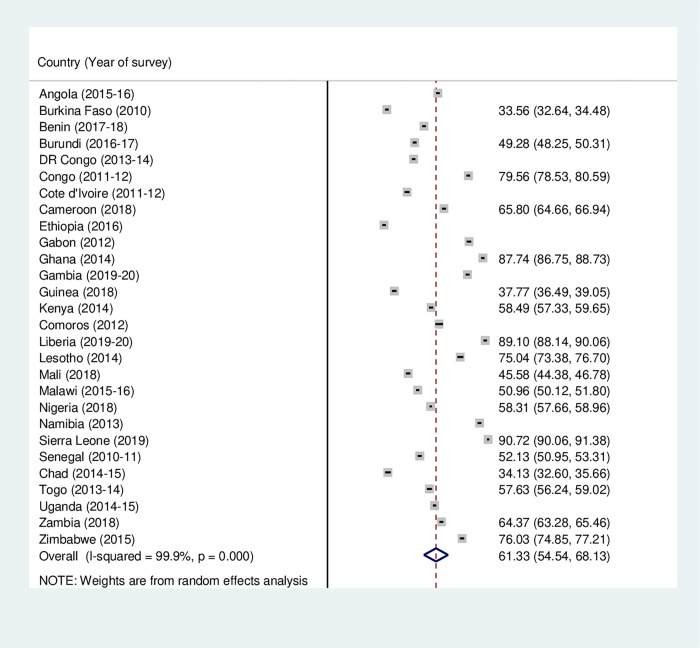
Forest plot showing the prevalence of four or more antenatal care visits among women in sub-Saharan Africa.

**Fig 2:**
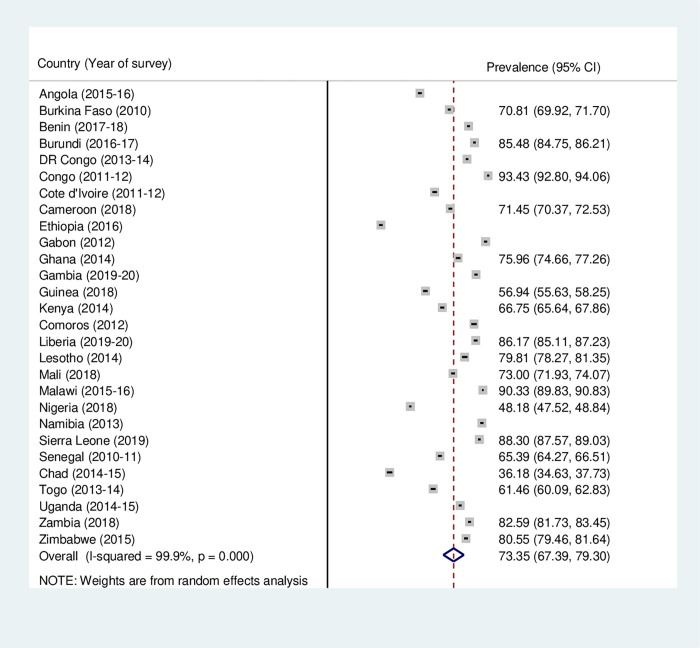
Forest plot showing the prevalence of skilled birth attendance during delivery among women in sub-Saharan Africa.

**Fig 3:**
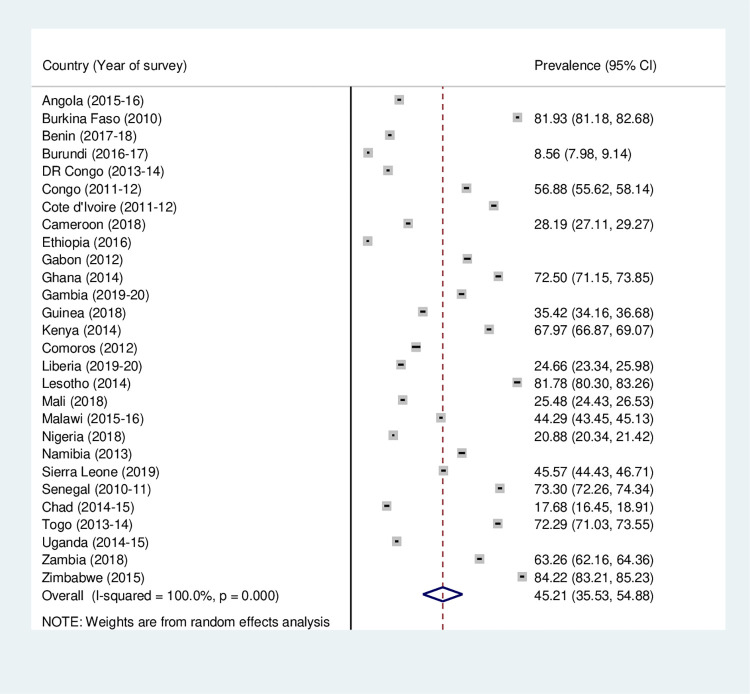
Forest plot showing the prevalence of postnatal care services utilization among women in sub-Saharan Africa.

We first extracted the data from the individual women’s files in the 28 countries and appended it for analysis. The data was cleaned, and all missing observations were dropped. Only the countries with the completed cases of variables of interest were included in the final analysis. First, percentages were used to present the results of the utilisation of the ANC, SBA, and PNC using a forest plot (Figs 1–3). We performed crosstabulation to determine the distribution of the outcome variables across the exposure variables and the covariates. Pearson’s chi-square test of independence was employed to determine the significant variables using the p-value (*p* < 0.05). We employed the ‘best subset variable selection method’ to obtain the variables for the regression analysis. According to Lindsey and Sheather [37], the best subset variable selection method when performed enables the researcher with the best combinations of predictors for each level of model complexity. To perform this, we used the Stata command ‘gvselect’ together with all the covariates to determine which set of covariates to include in the regression model. The output of the best selection methods included log-likelihood, and Akaike’s information criterion (AIC). We selected the set of variables with the lowest AIC for this study. To determine the influence of different types of mass media variables on ANC, SBA, and PNC, a multilevel logistic regression was adopted and modelled in three steps. Model 0, I, and II were fitted to include the outcome variable, key explanatory variables only, and key explanatory variables and covariates from the best selection method respectively. The rest of the AIC was used to test for the model fitness and comparison. Adjusted odds ratio (aOR) with their 95% confidence intervals (CIs) were used to present the results of the regression analysis in a tabular form. Furthermore, the intraclass correlation coefficient, and the variance component is reported. The women’s sample weight (v005/1,000,000) was applied in all analyses to alleviate biased estimates based on the DHS guidelines. Also, we used the survey set ‘svy’ command in Stata to adjust for the complex sampling technique employed by the DHS in all the analysis. Statistical significance was set at p-value less than 0.05. Stata software version 16.0 was used to perform the analysis.

### Ethical consideration

The study required no ethical clearance because the DHS dataset is freely available in the public domain. Prior permission to use the dataset was sought from the MEASUREDHS. We also adhered to ethical guidelines in the use of secondary dataset for publication. Detailed information about the DHS data usage and ethical standards are available at http://goo.gl/ny8T6X.

## Results and discussion

### Results

#### Prevalence of maternal health care service utilization among women in sub-Saharan Africa

Figs 1–3 outline the prevalence of maternal health care utilization among women in SSA. The study found that the prevalence of ANC, SBA and PNC utilization in SSA was 61.33% (95% CI: 54.54–68.13), 73.35% (95% CI = 67.39–79.30) and 45.21% (95% CI = 35.53–54.88), respectively. The lowest and highest prevalence of ANC utilization was recorded in Ethiopia (31.99%, [95% CI = 30.95–33.03]) and Sierra Leone (90.72%, [95% CI = 90.06–91.32]), respectively (Fig 1). Also, while Ethiopia recorded the least (31.08% [95% CI = 30.04–32.12) prevalence of SBA utilization, Congo had the highest (93.43%, [95% CI = 92.80–94.06]) (Fig 2). For PNC utilization, the prevalence ranged from (8.33%, [95% CI = 7.71–8.95]) in Ethiopia to (84.22%, [95% CI = 83.21–85.23]) in Zimbabwe (Fig 3).

#### Association between explanatory variables and maternal health care service utilization

Table 2 provides a detailed outline of the association between explanatory variables and the outcome variable. Exposure to mass media, maternal age (years), maternal educational level, marital status, religion, maternal current working status, parity, getting medical help for self, health insurance coverage, wealth index, and residence were significantly associated with ANC, all at *p* < 0.001. Also, at *p* < 0.001, exposure to mass media, maternal age (years), maternal educational level, marital status, religion, maternal current working status, parity, getting medical help for self, health insurance coverage, wealth index, and residence were significantly associated with SBA. Further, exposure to mass media, maternal age (years), maternal educational level, marital status, religion, parity, getting medical help for self, health insurance coverage, wealth index and residence were significantly associated with PNC, all at *p* < 0.001.

**Table 2:** Distribution of maternal health care service utilisation across explanatory variables.

Variables	Weighted N	Weighted %	Antenatal care		Skilled birth Attendance		Postnatal care	
			Yes	P-value	Yes	P-value	Yes	P-value
**Frequency of reading newspaper/magazine**				<0.001		<0.001		<0.001
Not at all	169389	85.1	55.4		68.3		38.6	
Less than once a week	16538	8.3	73.7		89.2		52.2	
At least once a week	13219	6.6	78.8		92.1		54.2	
**Frequency of listening to radio**				<0.001		<0.001		<0.001
Not at all	89951	45.2	51.7		64.3		33.1	
Less than once a week	38287	19.2	61.5		75.5		45.1	
At least once a week	70908	35.6	65.4		78.8		48.0	
**Frequency of watching television**				<0.001		<0.001		<0.001
Not at all	120949	60.7	50.3		63.3		35.4	
Less than once a week	23754	11.9	63.3		77.0		45.7	
At least once a week	54443	27.4	74.5		87.7		50.3	
**Maternal age (years)**				<0.001		<0.001		<0.001
15–19	14689	7.4	54.6		72.8		38.3	
20–24	44704	22.4	58.3		73.8		41.1	
25–29	51346	25.8	59.6		72.5		40.9	
30–34	40238	20.2	59.9		71.9		41.8	
35–39	29205	14.7	58.9		70.2		40.6	
40–44	14215	7.1	55.9		65.8		39.9	
45–49	4749	2.4	52.4		60.7		37.2	
**Maternal educational level**				<0.001		<0.001		<0.001
No education	75140	37.7	43.8		54.8		35.9	
Primary	61743	31.0	58.0		73.9		39.7	
Secondary	54351	27.3	75.0		88.6		47.1	
Higher	7912	4.0	87.9		96.5		50.7	
**Marital status**				<0.001		<0.001		<0.001
Never married	15137	7.6	68.3		83.9		44.3	
Married	138062	69.3	55.9		68.7		41.3	
Cohabiting	31703	15.9	64.3		76.5		36.0	
Widowed	2808	1.4	58.1		66.1		42.4	
Divorced	3299	1.7	58.0		74.1		45.3	
Separated	8137	4.1	61.0		79.7		39.9	
**Religion**				<0.001		<0.001		<0.001
Christianity	120804	60.7	63.1		78.3		41.0	
Islamic	69622	35.0	52.0		62.0		39.8	
African Traditional	3427	1.7	39.4		49.2		47.4	
No religion	4249	2.1	45.7		55.7		44.5	
Others	1045	0.5	61.5		74.5		37.0	
**Maternal current working status**				<0.001		<0.001		0.090
No	69199	34.7	56.3		68.8		40.2	
Yes	129947	65.3	59.6		73.1		41.0	
**Parity**				<0.001		<0.001		<0.001
1	41964	21.1	65.4		82.1		44.6	
2	37522	18.8	62.1		76.6		42.9	
3	32006	16.1	60.3		73.3		42.4	
4 or more	87654	44.0	52.9		63.8		37.4	
**Getting medical help for self: Permission to go**				<0.001		<0.001		<0.001
Not a big problem	159591	80.1	60.5		73.5		42.9	
Big problem	39555	19.9	50.0		63.9		32.1	
**Getting medical help for self: Distance to health facility**				<0.001		<0.001		<0.001
Not a big problem	119841	60.2	63.2		77.6		42.8	
Big problem	79305	39.8	51.3		62.5		37.6	
**Getting medical help for self: Getting money for treatment**				<0.001		<0.001		<0.001
Not a big problem	88982	44.7	64.9		77.5		43.9	
Big problem	110164	55.3	53.3		66.8		38.1	
**Health insurance coverage**				<0.001		<0.001		<0.001
No	186395	93.6	57.2		70.6		39.8	
Yes	12751	6.4	76.8		86.8		53.6	
**Wealth index**				<0.001		<0.001		<0.001
Poorest	42434	21.3	45.3		51.7		35.6	
Poorer	42292	21.2	51.6		61.7		37.9	
Middle	40013	20.1	57.7		72.0		40.6	
Richer	38963	19.6	65.0		83.6		43.5	
Richest	35444	17.8	76.0		93.7		47.4	
**Residence**				<0.001		<0.001		<0.001
Urban	70962	35.6	72.9		87.7		46.5	
Rural	128184	64.4	50.5		62.7		37.5	

*p-values obtained from Pearson’s Chi-square test.

#### Fixed and random effect results of the association between mass media exposure and maternal health care service utilization (ANC, SBA & PNC)

Table 3 shows the results of the multilevel mixed effect model analysis of the association between mass media exposure and ANC. Women who listened to radio at least once every week (aOR = 1.11, 95% CI = 1.07,1.15) were more likely to attend ANC as compared to those who did not listen to radio at all. Also, women who watched television at least once a week (aOR = 1.39, 95% CI = 1.33,1.46) were more likely to attend ANC as compared to those who did not watch television at all.

**Table 3:** Fixed and random effect results of the association between mass media and ANC.

Variables	Model 0	Model I aOR [95% CI]	Model II aOR [95% CI]
**Fixed-effect results**			
**Frequency of reading newspaper/magazine**			
Not at all		1 [Ref.]	1 [Ref.]
Less than once a week		1.69*** [1.61,1.79]	1.00 [0.95,1.06]
At least once a week		1.96*** [1.84,2.09]	1.05 [0.98,1.13]
**Frequency of listening to radio**			
Not at all		1 [Ref.]	1 [Ref.]
Less than once a week		1.18*** [1.14,1.23]	1.07*** [1.03,1.11]
At least once a week		1.22*** [1.18,1.26]	1.11*** [1.07,1.15]
**Frequency of watching television**			
Not at all		1 [Ref.]	1 [Ref.]
Less than once a week		1.45*** [1.39,1.51]	1.14*** [1.09,1.19]
At least once a week		2.28*** [2.18,2.39]	1.39*** [1.33,1.46]
**Random effects**			
PSU variance (95% CI)	0.38 [0.31, 0.46]	0.20 [0.16, 0.25]	0.10 [0.08, 0.12]
ICC	0.10	0.06	0.03
Wald chi-square	Reference	2893.75***	7430.28***
**Model fitness**			
Log-likelihood	-131721.12	-126674.48	-120309.31
AIC	263446.2	253365	240692.6
N	199146	199146	199146
Number of clusters	1611	1611	1611

aOR = adjusted odds ratios; 95% CI = 95% confidence intervals

* *p* < 0.05

** *p* < 0.01

*** *p* < 0.001

Ref. = Reference category; PSU = Primary Sampling Unit; ICC = Intra-Class Correlation coefficient; AIC = Akaike’s Information Criterion; N = total sample size.

Table 4 presents the results of the multilevel mixed effect model analysis of the association between mass media exposure and SBA utilization. Women who read newspaper/magazine at least once a week (aOR = 1.27, 95% CI = 1.14,1.41); listened to radio at least once a week (aOR = 1.12, 95% CI = 1.07,1.17); and watched television at least once a week (aOR = 1.32, 95% CI = 1.24,1.40), were more likely to utilize SBA than those who did not read newspaper/magazine; listen to radio; and watch television at all. Table 5 outlines the results of the multilevel mixed effect model analysis of the association between mass media exposure and PNC visits. The study found that women who read newspaper/magazine at least once a week (aOR = 1.35, 95% CI = 1.27,1.45); listened to radio at least once a week (aOR = 1.37, 95% CI = 1.32,1.42); and watched television at least once a week (aOR = 1.39, 95% CI = 1.32,1.47) were more likely to utilize PNC compared to those who did not.

**Table 4:** Fixed and random effect results of the association between mass media and SBA.

Variables	Model 0	Model I aOR [95% CI]	Model II aOR [95% CI]
**Fixed-effect results**			
**Frequency of reading newspaper/magazine**			
Not at all		1 [Ref.]	1 [Ref.]
Less than once a week		2.75*** [2.57,2.95]	1.24*** [1.15,1.34]
At least once a week		3.17*** [2.87,3.51]	1.27*** [1.14,1.41]
**Frequency of listening to radio**			
Not at all		1 [Ref.]	1 [Ref.]
Less than once a week		1.31*** [1.25,1.37]	1.18*** [1.13,1.24]
At least once a week		1.31*** [1.25,1.37]	1.12*** [1.07,1.17]
**Frequency of watching television**			
Not at all		1 [Ref.]	1 [Ref.]
Less than once a week		1.54*** [1.46,1.63]	1.10** [1.04,1.17]
At least once a week		3.01*** [2.84,3.19]	1.32*** [1.24,1.40]
**Random effects**			
PSU variance (95% CI)	0.68 [0.60, 0.78]	0.57 [0.49, 0.65]	0.67 [0.59, 0.76]
ICC	0.17	0.15	0.17
Wald chi-square	Reference	3699.89***	8599.89
**Model fitness**			
Log-likelihood	-113214.83	-106145.74	
AIC	226433.7	212307.5	188277
N	199146	199146	199146
Number of clusters	1611	1611	1611

aOR = adjusted odds ratios; 95% CI = 95% confidence intervals

* *p* < 0.05

** *p* < 0.01

*** *p* < 0.001; Ref. = Reference category; PSU = Primary Sampling Unit; ICC = Intra-Class Correlation coefficient; AIC = Akaike’s Information Criterion; N = total sample size.

**Table 5:** Fixed and random effect results of the association between mass media and PNC.

Variables	Model 0	Model I aOR [95% CI]	Model II aOR [95% CI]
**Fixed-effect results**			
**Frequency of reading newspaper/magazine**			
Not at all		1 [Ref.]	1 [Ref.]
Less than once a week		1.39*** [1.32,1.46]	1.25*** [1.19,1.32]
At least once a week		1.38*** [1.30,1.47]	1.35*** [1.27,1.45]
**Frequency of listening to radio**			
Not at all		1 [Ref.]	1 [Ref.]
Less than once a week		1.48*** [1.42,1.55]	1.29*** [1.23,1.35]
At least once a week		1.56*** [1.50,1.62]	1.37*** [1.32,1.42]
**Frequency of watching television**			
Not at all		1 [Ref.]	1 [Ref.]
Less than once a week		1.23*** [1.17,1.30]	1.14*** [1.08,1.20]
At least once a week		1.42*** [1.35,1.49]	1.39*** [1.32,1.47]
**Random effects**			
PSU variance (95% CI)	0.44 [0.38, 0.51]	0.39 [0.34, .45]	0.41 [0.36, 0.47]
ICC	0.12	0.11	0.11
Wald chi-square	Reference	1388.71***	2701.84***
**Model fitness**			
Log-likelihood	-130108.01	-127390.53	-124601.3
AIC	260220	2547797.1	249274.6
N	199146	199146	199146
Number of clusters	1611	1611	1611

Exponentiated coefficients; 95% confidence intervals in brackets; aOR adjusted odds ratios; CI Confidence Interval

* *p* < 0.05

** *p* < 0.01

*** *p* < 0.001

1 = Reference category; PSU = Primary Sampling Unit; ICC = Intra-Class Correlation; AIC = Akaike’s Information Criterion.

## Discussion

The study examined the association between frequency of mass media exposure and maternal health services utilization among women in SSA. The study found that the prevalence of ANC, SBA, and PNC utilization was 58.5%, 71.6%, and 40.7%, respectively. Variations in the prevalence of ANC, SBA and PNC utilization between countries were observed. The prevalence of ANC utilization was lowest in Ethiopia (32.0%) and highest in Sierra Leone (90.7%). Sierra Leone had a Free Health Care Initiative (FHCI) strategy, its effectiveness may lead to a significant increase in ANC service utilization among women [38]. In Ethiopia however, health extension workers have been trained to provide maternal health care including antenatal care to women but the progress of this initiative seem to be thwarted probably because these extension workers are not listed under skilled providers [39]. Also, while Ethiopia recorded the lowest (31.1%) prevalence of SBA utilization, Congo had the highest (93.4%). This finding in the case of Ethiopia could be a manifestation of the belief that SBA utilization is less salient and less considered by Ethiopian women [40]. PNC utilization prevalence ranged from 8.3% in Ethiopia to 84.2% in Zimbabwe. This finding may be as a result of some Ethiopian women practicing seclusion after delivery making them less likely to utilize PNC services [41–43]. For all the maternal health service utilization indicators, Ethiopia had the least prevalence, therefore, health policymakers in Ethiopia should take some insights from some of the countries that are doing well in this regard such as, Congo, Zimbabwe, and Sierra Leone. The suggestion also works for other SSA countries with relatively lower ANC utilization such as Burkina Faso (33.6%), Chad (34.1%), Guinea (37.8%), Cote d’Ivoire (45.0%), Mali (45.6%), DR Congo (48.7%), and Burundi (49.3%).

Similar to the observation of previous studies [4,22,44–46], this study found that women who listened to the radio at least once every week were more likely to have ANC visitations as against those who did not listen to the radio at all. A plausible account for this finding could be attributed to the substantial improvement in women’s awareness and the need to consider ANC uptake even if they intend having a home-based delivery [44,46]. Therefore, more maternal health service utilization programs targeted at radio listeners should be designed and implemented to help increase ANC uptake among women.

Also, women who watched television at least once a week were more likely to have ANC visitations compared to those who did not. Other studies [27,28,47,48] had similar findings. Women who watch television may have frequently been educated about the need to visit the health facility for ANC both for their health and that of the unborn child making them more likely to access ANC [27,28,47]. It could also be that the consequences of not having ANC visitations as experienced by other women that are shown on televisions may reduce women’s desire to neglect ANC visitations [27,28,47]. This finding underscores the need to increase the broadcasting of television-based maternal and health care utilization programs at regular times. For instance, more “tele-nurses” could be used to educate women on maternal health service utilization on television stations.

Women who read newspaper/magazine, listened to radio, and watched television at least once a week were more likely to utilize SBA than those who were not exposed to such media sources at all. This finding corroborates previous studies [27,28,47,49–51]. In recent times, newspapers, radio, and television are media outlets through which important health information is transmitted to women. In this light, women who utilize such media are easily accessible to information that would help them make informed decisions about their health, increasing their propensity to utilize maternal health services including SBA use [27,28,51]. Also, there is the likelihood that women who are exposed to mass media (radio, newspapers/magazines, and television) will have a positive attitude towards the use of maternal health services such as SBA as a result of what they have heard, watched, or read [47].

Similar to findings of some previous investigations [27,28,52–54], the study found that women who read newspaper/magazine, listened to radio, and watched television at least once a week were more likely to utilize PNC compared to those who were not exposed to such media sources at all. Reasonably, women who are exposed to mass media have a better knowledge of PNC services that certainly increase their likelihood of PNC uptake [52,55]. Women who are exposed to mass media (especially newspaper/magazine, radio and television) may have better behavioral intentions and or desire to utilize PNC compared to their counterparts who are not [54].

### Strengths and limitations

Nationally representative data among SSA countries were employed to assess mass media exposure and maternal healthcare services utilization in SSA. The study has offered insights into the importance of mass media on maternal healthcare services utilization. The wide coverage and rigor of the analytical procedure have enhanced the prospects of generalizing the findings to other contexts where maternal healthcare services utilization can be attained. However, due to the cross-sectional nature of the study design, causal inference cannot be drawn from current outcomes. The relationships established between the explanatory and outcome variables may vary over time. Recall bias, which is an intrinsic nature of cross-sectional data may lead to under-reporting of the events studied.

## Conclusion

The study identified a strong positive predictive relationship between mass media exposure and health services utilization. The study observed that exposure to radio and television were positively associated with ANC visitations. Moreover, exposure to mass media (newspaper/magazine, radio and television) were positively associated with SBA and PNC utilization. We, therefore, recommend that health policymakers and other non-governmental organizations should continuously invest resources in the design and implementation of maternal health service utilization educational programmes via all the mass media sources to scale up women’s maternal health service utilization uptake in SSA.
